# Molecular evolution of B_6 _enzymes: Binding of pyridoxal-5'-phosphate and Lys41Arg substitution turn ribonuclease A into a model B_6 _protoenzyme

**DOI:** 10.1186/1471-2091-9-17

**Published:** 2008-06-19

**Authors:** Rosa A Vacca, Sergio Giannattasio, Guido Capitani, Ersilia Marra, Philipp Christen

**Affiliations:** 1 Institute of Biomembranes and Bioenergetics, CNR, Via Amendola 165/A, I-70126 Bari, Italy; 2 Biochemisches Institut der Universität Zürich, Winterthurerstrasse 190, CH-8057 Zürich, Switzerland; 3 Paul Scherrer Institut, CH-5232 Villigen, Switzerland

## Abstract

**Background:**

The pyridoxal-5'-phosphate (PLP)-dependent or vitamin B_6_-dependent enzymes that catalyze manifold reactions in the metabolism of amino acids belong to no fewer than four evolutionarily independent protein families. The multiple evolutionary origin and the essential mechanistic role of PLP in these enzymes argue for the cofactor having arrived on the evolutionary scene before the emergence of the respective apoenzymes and having played a dominant role in the molecular evolution of the B_6 _enzyme families. Here we report on an attempt to re-enact the emergence of a PLP-dependent protoenzyme. The starting protein was pancreatic ribonuclease A (RNase), in which active-site Lys41 or Lys7 readily form a covalent adduct with PLP.

**Results:**

We screened the PLP adduct of wild-type RNase and two variant RNases (K7R and K41R) for catalytic effects toward L- and D-amino acids. RNase(K41R)-PLP, in which the cofactor is bound through an imine linkage to Lys7, qualifies for a model proto-B_6 _enzyme by the following criteria: (1) covalent linkage of PLP (internal aldimine); (2) catalytic activity toward amino acids that depends on formation of an imine linkage with the substrate (external aldimine); (3) adjoining binding sites for the cofactor and amino acid moiety that facilitate the transimination reaction of the internal to the external aldimine and stabilize the resulting noncovalent complex of the coenzyme-substrate adduct with the protein; (4) reaction specificity, the only detectable reactions being racemization of diverse amino acids and β-decarboxylation of L-aspartate; (5) acceleration factors for racemization and β-decarboxylation of >10^3 ^over and above that of PLP alone; (6) ribonuclease activity that is 10^3^-fold lower than that of wild-type RNase, attenuation of a pre-existing biological activity being indispensable for the further evolution as a PLP-dependent protoenzyme.

**Conclusion:**

A single amino acid substitution (Lys41Arg) and covalent binding of PLP to active-site Lys7 suffice to turn pancreatic ribonuclease A into a protein catalyst that complies with all plausible criteria for a proto-B_6 _enzyme. The study thus retraces in a model system what may be considered the committed step in the molecular evolution of a potential ancestor of a B_6 _enzyme family.

## Background

**Figure 1:**
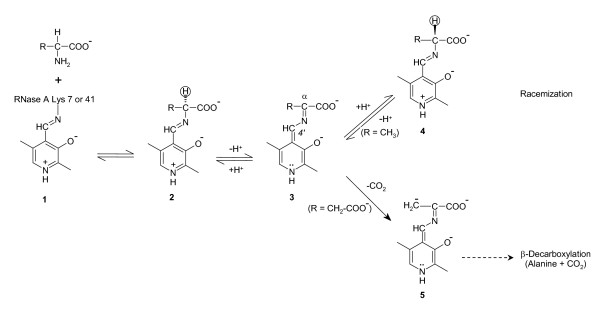
**Intermediates in protein-assisted PLP-dependent racemization and β-decarboxylation**. Only the reactions found to be catalyzed by the RNase-PLP adduct are depicted; not shown are the pathways for transamination, α-decarboxylation, Cα-Cβ cleavage, β/γ-elimination, and β/γ-substitution.

Pyridoxal 5'-phosphate (PLP), a derivative of vitamin B_6_, is one of the most versatile coenzymes and serves as prosthetic group of glycogen phosphorylase and numerous enzymes that catalyze manifold reactions of amino acids such as racemization, transamination, decarboxylation, aldol cleavage, as well as β- and γ-elimination and replacement reactions. In all PLP-dependent enzymes (B_6 _enzymes) that act on amino acid substrates the cofactor is bound through an imine linkage to the ε-amino group of an active-site lysine residue (internal aldimine **1**; Fig. 1). Hence, in all transformations of amino acids that are catalyzed by B_6 _enzymes the first covalency change is the same: through transimination the ε-amino group of the lysine residue is exchanged with the α-amino group of the amino acid substrate to form the planar external aldimine **2 **with its extended π system. The external aldimine intermediate may then proceed along different reaction pathways resulting in the manifold PLP-dependent transformations of amino acids. The cleavage of one of the bonds at Cα gives rise to the quinonoid intermediate **3**; the bond that is cleaved and the following covalency changes determine which particular transformation of the amino acid substrate is implemented (Fig. 1; for a concise review, see [1]). All reactions catalyzed by B_6 _enzymes are assumed to occur also, albeit very slowly, with amino acid substrates and PLP alone. The protein moiety of a given B_6 _enzyme modulates the intrinsic chemical disposition of the coenzyme-substrate adduct and determines which of the many possible pathways the adduct will adopt.

The B_6 _enzymes that act on amino acids belong to four independent evolutionary lineages [2-5]. The first step in the emergence of a B_6 _enzyme family very likely was the reaction of PLP with a lysine residue of a protoapoenzyme. The four progenitor B_6 _enzymes were selected among the available protoenzymes and, during a time period of at least 3000 million years, optimized their catalytic apparatus and specialized into the modern B_6 _enzymes with distinct reaction and substrate specificities and enormously increased catalytic efficiency [4,5]. In a previous attempt to generate *de novo *a PLP-dependent protein catalyst, PLP-dependent catalytic antibodies have been generated. In contrast to the modern B_6 _enzymes and their hypothetical protoenzymes, the catalytic antibodies, due to the immunization and selection protocol, do not bind PLP covalently [6-8].

Here we report an alternative approach to retracing the early steps in the evolution of B_6 _enzymes. We explored the catalytic capacity of a covalent adduct of a non-B_6 _protein with PLP. Before the advent of the technique of site-directed mutations, PLP has been widely used for lysine-specific chemical modification of proteins [9,10]. Bovine pancreatic ribonuclease A (RNase), which catalyzes the cofactor-independent endonucleolytic hydrolysis of RNA, was chosen for this study because its three-dimensional structure is known [11] and its physical, chemical and enzymic properties have been extensively investigated (for reviews, see [12-14]. The phosphate subsites interacting with the RNA substrate [15] endow the active site of RNase with high affinity for phosphate groups; RNase has indeed been reported to bind PLP covalently in a mutually exclusive fashion at the ε-NH_2 _group of either Lys7 or Lys41 [16-18] as well as to a lesser extent at the α-NH2 group of Lys1 [19]. We screened the PLP adduct of wild-type RNase and of two mutant RNases (K7R and K41R) with a single lysine residue at the active site for catalytic effects toward a series of L- and D-amino acids. RNase(K41R)-PLP showed catalytic properties as they might be expected from a PLP-dependent protoenzyme. It catalyzed with remarkable specificity the racemization of alanine and aspartate as well as the β-decarboxylation of L-aspartate. These reactions were at least three orders of magnitude faster than the corresponding unmeasurably slow reactions of the amino acids with PLP alone in the absence of RNase.

## Results

### RNase-PLP adduct

**Figure 2:**
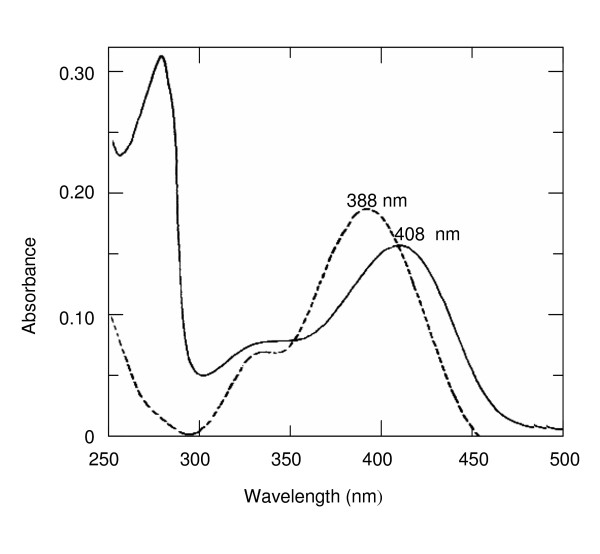
**Formation of internal aldimine between PLP and RNase**. (---) Absorption spectrum of 30 μM PLP in 50 mM 4-methylmorpholine pH 7.5 at 25°C; (—) spectrum upon addition of 30 μM of RNase.

**Figure 3:**
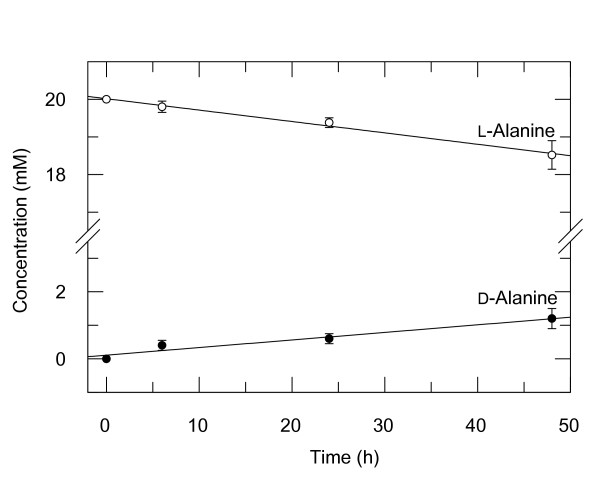
**Racemization of L-alanine by RNase-PLP adduct**. RNase-PLP (11 mM) was incubated with 20 mM L-alanine in 50 mM 4-methylmorpholine pH 7.5 at 25°C. Samples (20 μl) were withdrawn at the indicated times and analyzed by derivatization with Marfey reagent and subsequent HPLC (for details, see Materials and Methods). The mean values ± SE of 5 different experiments are shown. Similar data were obtained with D-alanine as substrate.

The aldimine adduct of PLP and either Lys41 or Lys7 of RNase formed immediately after mixing RNase and PLP at equimolar concentrations. The absorption maximum at 388 nm, typical of the free cofactor in aldehyde form [20], shifted to 408 nm (Fig. 2) as is characteristic for the aldimine of RNase with PLP [17]. Spectrophotometric determination of the concentration of PLP after dissociation of the adduct in 0.1 N NaOH (see Methods) showed that RNase bound PLP in a 1:1 molar ratio. Steric reasons preclude double aldimine formation at Lys7 and Lys41; at pH 6.0, PLP attaches to Lys41 or Lys7 in a 3:2 ratio [17]. The proximity of these lysine residues to a phosphate-binding subsite of the enzyme seems to be critical for aldimine formation; phosphate ions compete with PLP for binding to RNase [21]. The mixture of the aldimine adducts of RNase with PLP either at Lys41 or at Lys7 was analyzed for its capacity to catalyze PLP-dependent reactions of a series of neutral aliphatic, acidic, basic and aromatic amino acids. The mixed adducts were found to accelerate the racemization of alanine (Fig. 3) and at a slightly slower rate of dicarboxylic and aromatic amino acids. β-Decarboxylation of L-aspartate to L-alanine also took place, whereas no β-decarboxylation of D-aspartate was detected (Table 1). No transaminase activity, i.e. formation of pyridoxamine-5'-phosphate (PMP), was detected by spectrophotometry (see Methods) with any of the tested substrates. Although the rates of the PLP-dependent reactions that were catalyzed by RNase-PLP were slow, they by far (>10^3^) exceeded those of the unmeasurably slow control reactions without RNase; neither consumption of substrate nor formation of products were detected after incubation of all tested amino acids with PLP in the absence of RNase with the exception of the racemization of L- and D-glutamate (Table 1). Likewise, no amino acid transformations were detected in controls containing amino acids and RNase without PLP. Reduction of the internal aldimine bond [17] completely and irreversibly abolished the PLP-dependent catalytic activities of RNase indicating that they require the formation of covalent coenzyme-substrate adducts (Fig. 1). The reduction of the internal aldimine bond of the RNase-PLP adduct was monitored by the disappearance of the absorption maximum of the aldimine at 408 nm and the appearance of an absorption peak at 325 nm.

**Table 1:** Rate constants of PLP-dependent reactions catalyzed by the wild-type RNase-PLP adduct^a^

Substrate^*b*^	Reaction^*c*^	k_obs _× 10^7*d *^	
		(s^-1^)	
		
		RNase-PLP	Free PLP^*e*^
L-Alanine	Racemization	5.3 ± 2.0	< 0.01
D-Alanine	Racemization	6.5 ± 1.0	< 0.01
L-Glutamate	Racemization	1.2 ± 0.7	0.1 ± 0.05
D-Glutamate	Racemization	3.1 ± 2.2	0.1 ± 0.05
L-Aspartate	Racemization	3.3 ± 0.5	< 0.01
	β-Decarboxylation	2.2 ± 0.5	< 0.01
D-Aspartate	Racemization	2.2 ± 0.7	< 0.01
L-Phenylalanine	Racemization	3.1 ± 1.2	< 0.01
D-Phenylalanine	Racemization	2.2 ± 1.0	< 0.01

^*a *^The concentration of RNase-PLP and free PLP was 11 mM; the substrate concentration was 20 mM. The assays were run at pH 7.5 and 25°C. For details of the analytical protocols, see Methods.

^*b *^Activities were tested and found below detection level (see Footnote *e*) with L-serine, D-serine, L-serine-*O*-sulfate, L-cysteine sulfinate, L-valine, L-norvaline, L-leucine, L-norleucine, L-isoleucine, L-2-aminoadipate, 5-aminovalerate as substrates.

^*c *^No transamination reaction was detectable with any of the amino acids analyzed.

^*d *^The k_obs _value denotes the rate of the reaction (μmol·s^-1^) under the given conditions per μmol RNase-PLP adduct or PLP, respectively. The results are the mean ± SE of at least four independent measurements.

^*e *^Neither substrate consumption nor reaction products were detected with the Marfey assay after 336-h incubation of L- and D-alanine and L- and D-phenylalanine or after 24-h incubation of L- and D-aspartate with the free cofactor (25 mM). Experiments with the L- or D-aspartate/oxalacetate pair could not be continued for more than 24 h because of the inherent instability of oxalacetate [28]. In our experimental set-up, the detection limit for a reaction was of the order of 10^-9 ^s^-1 ^with the Marfey assay and 10^-8 ^s^-1 ^in the spectrophotometric assay for transamination (see Methods).

### Recombinant wild-type and variant RNase-PLP adducts

To eliminate the ambiguity of PLP attaching either to Lys7 or Lys41 and possibly to improve the PLP-dependent catalytic activity of RNase-PLP, Lys7 and Lys41 were separately replaced by an arginine or alanine residue. Recombinant wild-type and the four mutant RNases (K41R, K41A, K7R, K7A) were purified from *E. coli *cells to homogeneity, and their ribonuclease activity was measured (Table 2). K41R and K41A retained only 0.1% and 0.01%, respectively, of the ribonuclease activity of the wild-type enzyme. Lys41 has indeed been reported to participate directly in the catalytic mechanism of ribonuclease [22]. In contrast to the K41 variants, K7R and K7A retained 98% and 22%, respectively, of the wild-type RNase activity.

**Table 2:** Catalytic activities of recombinant wild-type and mutant RNases.

	k_cat_^*a *^	k_obs_^*b *^		
	(s^-1^)	(s^-1^)		
		
RNase-PLP	Ribonuclease activity (tRNA)	Racemization (L-Alanine)	Racemization (L-Aspartate)	β-Decarboxylation (L-Aspartate)
		
Wild-type^*c*^	36 ± 1.0	(6 ± 1.0) × 10^-7^	(3 ± 0.5) × 10^-7^	(2 ± 0.2) × 10^-7^
K7R	35.6 ± 1.0	(6 ± 0.5) × 10^-7^	(7 ± 0.7) × 10^-7^	< 10^-9*d*^
K41R^*c*^	0.036 ± 0.002	(2 ± 0.5) × 10^-6^	(3 ± 0.5) × 10^-7^	(4 ± 0.5) × 10^-6^
K7A^*e*^	8 ± 0.5	-	-	-
K41A^*e*^	0.0034 ± 0.0002	-	-	-

^*a*^Ribonuclease activity was measured as described [41]; for details, see Methods. The ribonuclease activity of the recombinant wild-type and mutant RNases in the absence of PLP was comparable to that of the corresponding RNase-PLP complexes.

^*b *^The k_obs _value denotes the rate of the reaction (μmol·s^-1^) under the given conditions per μmol RNase-PLP adduct. PLP-dependent activities were measured at pH 7.5 and 25°C; the concentration of the RNase-PLP adduct was 11 mM; the substrate concentration was 20 mM (for details, see Methods). Results are the mean ± SE of three independent measurements. The rates of the PLP-dependent reactions in the absence of RNase were below the detection limit of 10^-9 ^s^-1^.

^*c *^These enzymes catalyze the racemization of L-phenylalanine with a rate of (3 ± 0.4) × 10^-7 ^s^-1^.

^*d *^Detection limit for a reaction with the Marfey assay is 10^-9^s^-1^.

^*e *^These RNase variants showed low affinity for PLP (see text), their PLP-dependent activities have not been measured.

To assess the binding affinity of wild-type and variant RNases for PLP, RNase and PLP were mixed at equimolar concentration. After size-exclusion chromatography, the wild-type protein, RNase(K41R) and RNase(K7R) showed an absorption maximum at 408, 400 and 396 nm, respectively, as is characteristic of the aldimine adduct. The PLP/RNase molar ratio was determined after dissociation of PLP from the protein with NaOH (see Methods) and was found to be 1.0 for the wild-type enzyme and K7R, and 0.8 for K41R. In contrast, K7A showed low absorbance intensity (λ_max _396 nm) with a PLP/RNase molar ratio of only 0.2, and K41A did not show any absorbance in the wavelength range of the coenzyme. Only the Lys → Arg mutants, which had retained the high affinity for PLP, were screened for PLP-dependent catalytic activities (Table 2).

**Figure 4:**
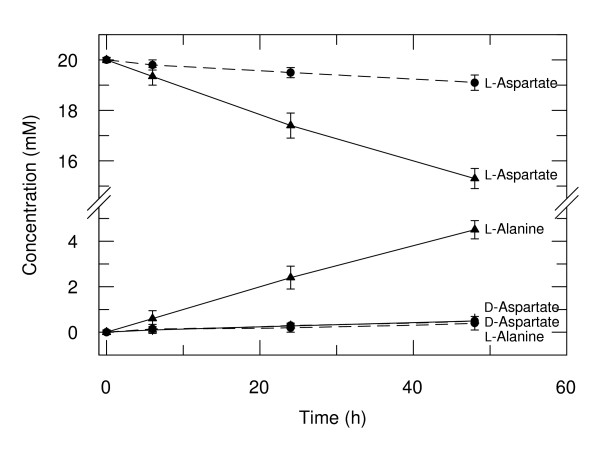
**β-Decarboxylation and racemization of L-aspartate by RNase(K41R)-PLP**. Recombinant RNase(K41R)-PLP (▲, —) and wild-type RNase-PLP (●, --) were incubated with 20 mM L-aspartate in 50 mM 4-methylmorpholine pH 7.5 at 25°C. The concentration of RNase-PLP adduct was 11 mM in the case of the wild-type enzyme and 7 mM in the case of K41R. Samples of 20 μl were withdrawn at the indicated times and analyzed by derivatization with Marfey reagent and subsequent HPLC as described under Materials and Methods. The mean values ± SE of 5 different experiments with standard deviations are shown.

The PLP-dependent catalytic properties of recombinant wild-type RNase proved essentially the same as those of the enzyme isolated from bovine pancreas. However, the PLP-dependent catalytic activities of K41R were higher than those of wild-type RNase. With K41R-PLP the β-decarboxylation of L-aspartate to L-alanine (Fig. 4) and the racemization of alanine were 20 and 3 times faster, respectively, than with the wild-type enzyme; its ribonuclease activity, however, was three orders of magnitude lower than that of wild-type RNase-PLP (Table 2). The aspartate β-decarboxylase/ribonuclease activity ratio and the alanine racemase/ribonuclease activity ratio of K41R-PLP thus are 2 × 10^4 ^times and 3300 times, respectively, higher than those of wild-type RNase-PLP. In this context, it should be noted that the covalent binding of PLP to RNase is reversible and does not impair its ribonuclease activity (Footnote *a *in Table 2). In the case of RNaseK7R-PLP, the ribonuclease and racemase activities are about the same as those of the wild-type enzyme, whereas the β-decarboxylase activity is more than two orders of magnitude lower.

**Figure 5:**
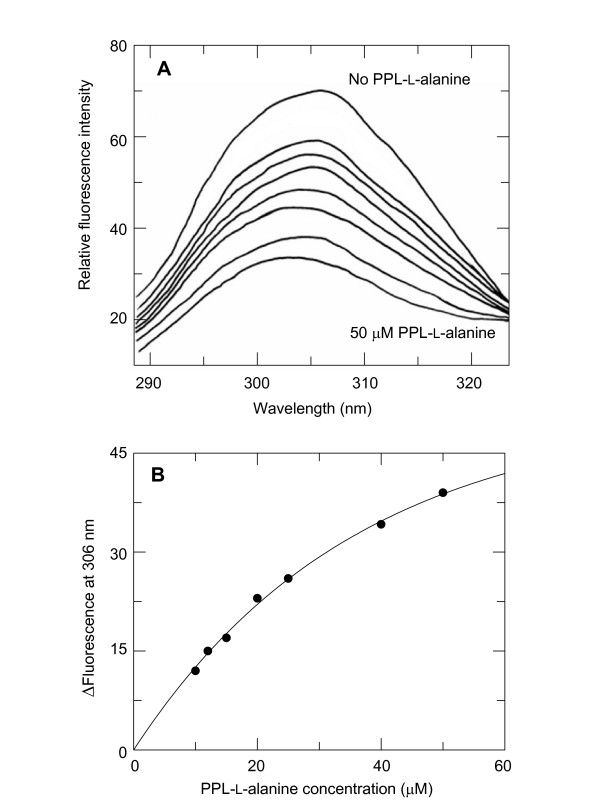
**Fluorescence titration of RNase(K41R) with PPL-L-alanine**. Fluorescence was measured with 5 μM RNase(K41R) in 50 mM 4-methylmorpholine pH 7.5 at 25°C. PPL-L-alanine concentrations were 10, 12, 15, 20, 25, 40 and 50 μM. **A**, Fluorescence spectra; **B**, nonlinear regression analysis gives a *K*'_d _value of 23 μM. Bovine RNase A contains six tyrosine residues and no tryptophan residue [45].

The binding affinities of wild-type RNase and K41R for PLP, PMP and both enantiomers of *N*^α^-(5'-phosphopyridoxyl)-alanine (PPL)-alanine were determined by measuring the quenching of the intrinsic fluorescence upon addition of the ligand (Fig. 5 shows the fluorescence titration of RNaseK41R with PPL-L-alanine). The *K*_*d *_values of wild-type and mutant RNase proved to be similar and showed a clearly higher affinity for both enantiomers of PPL-alanine than for PLP and PMP (Table 3). Apparently, both wild-type RNase and K41R interact not only with the coenzyme but also with the substrate moiety of the coenzyme-substrate adduct.

**Table 3:** Dissociation equilibrium constant of wild-type RNase and K41R for PLP, PMP, PPL-L-alanine and PPL-D-alanine

	*K*^'^_d_*^a^*	
	(μM)	
	
Ligand	Wild-type	K41R
PLP	120 ± 5	140 ± 10
PMP	106 ± 5	107 ± 3
PPL-L-alanine	23 ± 1	23 ± 1
PPL-D-alanine	45 ± 1	40 ± 3

^*a *^The constants were determined by the quenching of the intrinsic fluorescence of RNase at pH 7.5 upon addition of the ligand (see Methods) as shown in Fig. 5 for RNase(K41R) and PPL-L-alanine. Results are the mean ± SE of three independent measurements.

### Molecular modeling of RNase(K41R)-PLP

**Figure 6:**
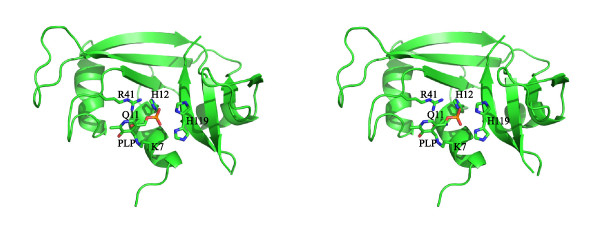
**Stereo cartoon representation of RNase(K41R)-PLP**. The cofactor and selected binding residues appear in stick mode, in green and atom colors. His119 is shown in two alternative conformations, which are also seen in the template structure [24].

The simulation of the internal aldimine of RNase(K41R) with PLP was based on a crystal structure of RNase containing a phosphate ion bound in the active site (PDB entry code 1SSC; [23]). The covalent aldimine bond between PLP and Lys 7 of K41R together with the anchoring of the phosphate group to specific amino acid residues allowed to identify with good confidence the region where the cofactor binds to RNase (Fig. 6). The positioning of the cofactor was based on a crystallographic study of three wild-type RNase derivatives with PLP [24]. Derivative B in that study contained PLP bound to Lys7 of wild-type RNase. Due to unfortunate circumstances, the coordinates of that structure are not available (Dr. M. Vilanova, personal communication). Therefore, careful visual inspection of Figure 2 (stereo) in [24] was employed to place PLP in a corresponding position in our RNase(K41R)-PLP model. Coordinate reconstruction from the stereo figure was not attempted since Figure 2 in [24] shows three superimposed structures. According to our model, Arg41 contributes to the binding of the phosphate group of PLP. There appears to be no acidic residue that could form a salt bridge with the pyridinium nitrogen of the cofactor ring. Spectroscopic measurements suggest that the cofactor in PLP-Lys7-RNase, in which PLP is attached to the same lysine residue as in K41R-PLP, is mainly solvent-exposed [25]. Our model is in accord with this observation.

## Discussion

In the emergence of a B_6 _protoenzyme, i. e. of a potential progenitor of one of the four B_6 _enzyme families, the pivotal events conceivably were on the one hand the association with PLP that endowed the prospective protoapoenzyme with an entirely new catalytic potential and on the other hand mutations that improved the PLP-dependent catalytic activities as well as attenuated a possibly preexisting catalytic activity. Attenuation and eventual loss of a preexisting activity allows the evolutionary pressure exerted upon the protein to shift to its newly acquired catalytic function. If the original activity was essential for the cell, preceding gene duplication had, of course, to procure two separate gene products, one maintaining the original function, the other becoming a B_6 _protoenzyme. RNase(K41R)-PLP corresponds to a plausible experimental reconstruction of a protein that has passed such a crossroads of molecular evolution.

### Criteria for a B_6 _protoenzyme

The mechanism of PLP-assisted transformation of amino acids [1] and the course of the molecular evolution of B_6 _enzymes [4,5] suggest that a PLP-dependent protoenzyme was equipped with the following features, all of which are found in RNase(K41R)-PLP:

(i) The cofactor is covalently attached to the ε-amino group of a lysine residue. The formation of the internal aldimine **1 **(Fig. 1) is found in all B_6 _enzymes catalyzing reactions of amino acids. A number of observations argue for the ubiquitous internal aldimine being an evolutionary trait rather than a mechanistic necessity [4]. The most weighty evidence for this notion is that the same PLP-lysine linkage is also found in glucan phosphorylases, in which the aldehyde group of PLP does not at all interact with the substrate [26]. The encounter of PLP with a candidate protoapoenzyme must have been an infrequent event given the likely very low concentration of either reactant. Covalent binding of the cofactor prevented its rapid loss, protected the reactive aldehyde group against side reactions, and thus probably was of decisive selective advantage in the emergence of B_6 _enzymes (for a more detailed discussion, see [4]).

(ii) The formation of the internal aldimine **1 **implicates that the first covalent PLP-amino acid adduct, the external aldimine **2**, is formed through transimination (Fig. 1) rather than *de novo*. In RNase(K41R), the affinity of which for both enantiomers of PPL-alanine is higher than that for PLP and PMP, a low-affinity binding site for the amino acid substrate adjacent to the PLP-binding site apparently facilitates the transimination step. The complete loss of catalytic activity upon reduction of the internal imine bond with borohydride documents that transimination is as indispensable in RNase(K41R)-PLP as it is in B_6 _enzymes.

(iii) Transimination abolishes the covalent bond between cofactor and proto-enzyme (Fig. 1), requiring noncovalent interactions to retain the coenzyme-substrate adduct at the active site. RNase(K41R)-PLP also fulfills this precondition.

(iv) The reaction specificity of RNase(K41R)-PLP is well defined. The evolutionary pedigrees of the four known B_6 _enzyme families operating in the metabolism of amino acids show that the protoenzymes first diverged into reaction-specific catalysts, which then branched further and acquired substrate specificity [4,5]. The only reactions catalyzed by RNase(K41R)-PLP at measurable rate are the racemization of amino acids and the β-decarboxylation of L-aspartate. The assay with Marfey reagent [27,28] allows to exclude any other potential reaction of the adduct of PLP with the tested amino acids (Fig. 1). The joint occurrence of racemization and β-decarboxylation may be explained by the similarity of the two reaction pathways, which have in common the deprotonation at Cα of the external aldimine **2 **producing the quinonoid intermediate **3**. In the case of L-amino acid substrates, the deprotonation of the aldimine occurs from its *re *side. The racemization of L-aspartate as catalyzed by K41R-PLP is by one order of magnitude slower than the β-decarboxylation of the same substrate. The rate-limiting step in the racemization of L-aspartate thus seems to be the reprotonation of the quinonoid intermediate **4 **from the *si *side to give D-aspartate. The substrate specificity of K41R-PLP, as expected from a B_6 _protoenzyme, is less strictly defined than its reaction specificity; in addition to alanine, which is the best substrate, acidic and aromatic amino acids are accepted as substrates. The substrate and reaction specificity of B_6 _enzymes may be changed by substitution of critical active-site residues [29-33].

(v) The acceleration factors that are due to the protein moiety of RNase(K41R)-PLP, i.e. the factors, by which the rates of the RNase(K41R)-PLP-catalyzed reactions exceed the rates of the PLP-catalyzed reactions in the absence of RNase(K41R), are >10^3 ^for both the racemization of alanine and the β-decarboxylation of L-aspartate (Table 2). Only minimum values of the acceleration factors can be given, because in the presence of PLP alone the rates of both reactions are unmeasurably slow (for the detection limits of the assays, see Table 1, Footnote *e*). The catalytic contribution of the cofactor can only be approximately assessed; previous experiments have given an estimate of the acceleration factor due to PLP in the absence of a protein catalyst of about 10^4 ^for the β-elimination reaction of β-chloroalanine and the transamination reaction [6]. The conjoint acceleration factor of RNase(K41R)-PLP may thus be assumed to be >10^7^.

### Mechanistic aspects

The B_6 _enzymes that act on amino acid substrates invariably utilize the active-site lysine residue that covalently binds PLP as the general base group abstracting the proton from Cα(3). Molecular modeling shows that such a role for Lys7 is highly improbable in RNase(K41R)-PLP, in which one face of PLP is fully and the other partly accessible to the solvent. Most likely, bulk water molecules deprotonate the coenzyme-substrate adduct at Cα and, in the racemization reaction, reprotonate it from the opposite side.

The previously generated PLP-dependent catalytic antibody 15A9 [6,7], the high-resolution crystal structure of which has recently been determined [8], does not possess a PLP-binding active-site lysine residue. Nevertheless, antibody 15A9 catalyzes the transamination reaction of D-alanine and other hydrophobic amino acids with PLP and is by 3 orders of magnitude catalytically more active than RNase(K41R). The handicap of RNase(K41R)-PLP *vs *the catalytic antibody might be a less favorable positioning of PLP and the amino acid substrate for forming a planar coenzyme-substrate adduct. Another reason for the relatively slow reactions might be an orientation of the Cα-H bond that is unfavorable for deprotonation. In B_6 _enzymes, the bond to be broken invariably lies, together with the Cα-N bond, in a plane orthogonal to the plane of the coenzyme-imine π system [3,34]. This conformation corresponds to minimum free energy of the transition state for bond cleavage, as it allows maximum σ-π overlap between the bond to be broken and the pyridine-imine system.

For improving the efficacy of RNase(K41R) as a PLP-dependent protein catalyst directed molecular evolution would seem to be the method of choice. Previous studies using forced evolution have succeeded to change the substrate specificity of B_6 _enzymes. Repeated rounds of metabolic panning of randomly mutagenized bacterial aspartate aminotransferase increased its activity toward branched-chain amino acids by a factor of 10^6^, with, remarkably, only a single residue out of a total of 17 mutagenized residues interacting directly with the substrate [35]. Similarly, forced evolution replacing 13 amino acid residues endowed aspartate aminotransferase with tyrosine aminotransferase activity that was two orders of magnitude higher than that of the parent wild-type enzyme [36].

## Conclusion

RNase(K41R)-PLP fully complies with all reasonable criteria for a model B_6 _protoenzyme. Conceivably, the ancestor proteins of each of the four B_6 _enzyme families were selected from proteins with properties similar to those of RNase(K41R). The selection criteria very likely included the same constraints that guided the choice of RNase(K41R) as model B_6 _protoenzyme and were considered in the selection of antibody 15A9 [6,7]. The existence of four B_6 _enzyme families of entirely different fold [2-5] indicates that the structural requirements for efficient protein-assisted pyridoxal catalysis can be met with quite different protein scaffolds. Conversely, comparison of enzyme structures shows that in numerous instances one and the same protein fold renders entirely different catalytic functions, e. g. the enzymes of the alanine racemase family of B_6 _enzymes are (βα)_8 _proteins and remotely but definitely related to other enzymes with quite diverse non-PLP-dependent catalytic activities [4,37].

The present study propounds RNase(K41R) as a plausible model B_6 _protoenzyme; its evolutionary potential was, however, not tested. Future attempts to improve the catalytic performance of RNase(K41R)-PLP may be expected to contribute to a deepened appreciation of the interplay of chance and chemical-mechanistic necessity during the molecular evolution of PLP-dependent enzymes.

## Methods

### Site-directed mutagenesis of RNase, expression and purification of recombinant wild-type and mutant RNase

The cDNA sequence [38] encoding bovine pancreatic RNase A (E.C. 3.1.27.5) was inserted into the NdeI/HindIII sites of the expression vector pET22b(+) (Novagen, Darmstadt, Germany). The recombinant plasmid pET22b/RNase A was used for oligonucleotide-directed mutagenesis according to Kunkel [39]. The synthetic oligonucleotides CGT CGT CGG GCT AAA CTC GCC, CGT CGT CGG CGT AAA CTC GCC, CTA GCT ACG GCT GGT CAC TTG and CTA GCT ACG CGT GGT CAC TTG were used for the K7R, K7A, K41R and K41A amino acid substitutions, respectively. The mutations were confirmed by determination of the nucleotide sequences on both strands. Wild-type and mutant enzymes were expressed in the *E. coli *strain BL21 (Novagen, Darmstadt, Germany) and purified essentially as described [40]. The maximum yield was 60 mg pure recombinant enzyme per liter of culture medium. The purified enzymes were stored at -20°C in 50 mM 4-methylmorpholine pH 7.5 at a concentration of 100–200 mg/ml. Ribonuclease activity was determined by following the changes in A_300_ with yeast RNA (Sigma, Milan, Italy) as substrate (0.5 mg/ml) in 0.1 M sodium acetate pH 5.0 [41].

### Preparation of RNase-PLP adduct

A solution of bovine pancreatic RNase A (type II from Sigma, Milan, Italy) or recombinant wild-type and mutant RNase at a concentration of 10–50 mg/ml in 50 mM 4-methylmorpholine pH 7.5 was incubated at 25°C in the dark for 15 min with a 5-fold molar excess of PLP. Excess PLP was removed through Sephadex G-25 chromatography. The protein-containing fractions were pooled, concentrated and stored frozen at -20°C in the dark at a protein concentration of about 20 mM. The RNase-PLP adduct was stable under these conditions for at least one month; in solution at 25°C it proved stable for at least 48 h. RNase concentration was calculated from the absorbance at 278 nm with a molar absorption coefficient of 9800 M^-1 ^cm^-1 ^[42]. PLP concentration was calculated from the absorbance at 388 nm using a molar absorption coefficient of 4900 M^-1 ^cm^-1 ^at neutral pH [20]. The extent of modification of RNase with PLP was determined from the absorbance at 388 nm of free PLP after dissociation of the RNase-PLP adduct in 0.1 N NaOH using a molar coefficient of 6600 M^-1 ^cm^-1 ^[20]. The PLP-lysine aldimine double bond in the RNase-PLP adduct was reduced by adding a freshly prepared solution of NaBH_4 _(final concentration 2 mM) at room temperature [17]. Immediately after bleaching of the solution, excess NaBH_4 _was separated from the reduced enzyme by Sephadex G-25 chromatography.

### Assay for detection of PLP-dependent transformations of amino acids

RNase-PLP adduct (11 mM) was incubated in 50 mM 4-methylmorpholine pH 7.5 at 25°C in the dark with the amino acid substrate (20 mM). The respective assays with free PLP (11 or 25 mM) instead of the RNase-PLP adduct were performed in the presence of the cognate oxo acids (10 mM or 25 mM, respectively) to reverse the potential transamination of PLP to catalytically inert PMP [6]. The solutions were 0.2-μm filtrated. Samples of 20 μl were withdrawn at different times during a period of 48 h. For determination of L-and D-amino acids and of amines as decarboxylation products, samples of the reaction mixture were deproteinized by centrifugation at 12,000 g for 30 min with Microcon 3 ultrafiltration devices (Millipore), derivatized with Marfey reagent [27] and analyzed by reverse-phase HPLC [29]. Newly generated peaks were identified and quantified by comparison with reference substances. The assay thus detects both the consumption of amino acid substrate and the formation of any product, including the enantiomer of the substrate, as long as the product carries a primary amino group [27,28].

For measuring transaminase activity, samples were withdrawn at intervals during a period of 48 h and analyzed for a decrease in A_408 _(wild-type enzyme), A_400 _(K41R) or A_396 _(K7R), reflecting a decrease in protein-bound PLP, as well as for an increase in A_325 _to detect PMP as transamination product. In the assay for serine dehydratase activity, samples of the reaction mixture were deproteinized as above and directly analyzed for pyruvate with lactate dehydrogenase and NADH [30].

### Determination of dissociation equilibrium constants

The *K*'_d _values of RNase for PLP, PMP and PPL-L-alanine and PPL-D-alanine, prepared by reduction of the PLP-amino acid adducts with sodium borohydride [6], were determined in 50 mM 4-methylmorpholine pH 7.5 at 25°C by measuring the quenching of the intrinsic fluorescence of the protein (excitation wavelength 279 nm; wavelength of maximum emission 306 nm). The concentration of the enzyme was 5 μM; the concentrations of the PLP, PMP and PPL-amino acids were in the range of 10–200 μM. *K*'_d _values were calculated by nonlinear regression analysis.

### Molecular modeling

A molecular model of the internal aldimine of RNase(K41R) with PLP was built based on a crystal structure of wild-type RNase containing a phosphate ion (PDB code 1SSC, Ref. [23]) and on a study of three wild-type RNase-PLP adducts, among which one contained PLP covalently attached to Lys7 of wild-type RNase [24]. Lys41 was mutated to arginine with the program *Coot *[43], and the position of its side chain manually optimized for binding the phosphate group of PLP. The cofactor (positioned as in Ref. [24]) was mainly solvent-exposed, with one face fully exposed and the other partly accessible, in agreement with previous spectroscopic measurements [25]. In a final step, the model was energy-minimized with *Phenix *[44]. In this phase, the geometry and stereochemistry of the imine bond between the cofactor and Lys7 were optimized.

## Abbreviations

PLP: pyridoxal-5'-phosphate; B_6 _enzyme: vitamin B_6 _(PLP)-dependent enzyme; PMP: pyridoxamine-5'-phosphate; PPL-: *N*^α^-(5'-phosphopyridoxyl)-.

## Authors' contributions

PC conceived and coordinated the study; RAV and SG designed and performed the experimental work; GC designed and performed molecular modeling; EM participated in design and coordination of the study; RAV, SG, GC and PC wrote the manuscript. All authors read and approved the final manuscript.
